# Time-Dependent Expression of Arc and Zif268 after Acquisition of Fear Conditioning

**DOI:** 10.1155/2010/139891

**Published:** 2010-05-26

**Authors:** Mary E. Lonergan, Georgette M. Gafford, Timothy J. Jarome, Fred J. Helmstetter

**Affiliations:** Department of Psychology, University of Wisconsin-Milwaukee, 2441 E. Hartford Avenue, Milwaukee, WI 53211, USA

## Abstract

Memory consolidation requires transcription and translation of new protein. Arc, an effector immediate early gene, and zif268, a regulatory transcription factor, have been implicated in synaptic plasticity underlying learning and memory. This study explored the temporal expression profiles of these proteins in the rat hippocampus following fear conditioning. We observed a time-dependent increase of Arc protein in the dorsal hippocampus 30-to-90-minute post training, returning to basal levels at 4 h. Zif268 protein levels, however, gradually increased at 30-minute post training before peaking in expression at 60 minute. The timing of hippocampal Arc and zif268 expression coincides with the critical period for protein synthesis-dependent memory consolidation following fear conditioning. However, the expression of Arc protein appears to be driven by context exploration, whereas, zif268 expression may be more specifically related to associative learning. These findings suggest that altered Arc and zif268 expression are related to neural plasticity during the formation of fear memory.

## 1. Introduction

A predominant question in neuroscience is how memory functions are supported by the central nervous system and what cellular processes are necessary. One focus of this research is on protein-dependent synaptic modifications that occur as a consequence of neuronal activity. Signaling cascades activated at the time of learning can induce the transcription of particular genes, ultimately leading to *de novo* protein synthesis and subsequent structural changes to support long-term memories.

Gene expression plays a critical role in these postactivation changes in neurons. Immediate-early genes (IEGs) are induced soon after neuronal activity, and they participate in diverse functions. Some IEGs are regulatory transcription factors (e.g., zif268/Egr1) responsible for inducing transcription of late-response genes, while others are effector IEGs (e.g., Arc/Arg3.1) that are directly involved in cellular changes at locations such as the cytoskeleton or receptors. Many IEGs are translated in the soma. However, the transcripts of some IEGs, such as activity-regulated cytoskeleton-associated protein (Arc), are transported to the dendrites and protein synthesis occurs there [1], thus making Arc a reasonable target for researchers investigating the underlying mechanisms of postsynaptic changes supporting memory formation.

Arc (also called Arg3.1) is a plasticity-related gene whose induction occurs soon after synaptic activation [2–4], mRNA transcription is independent of *de novo* protein synthesis [3], and expression is primarily in excitatory neurons following behavioral experience [5]. Arc contains a synaptic activity-responsive element (SARE) in the promoter upstream of the initiation site, which is necessary for transcription and sufficient for the induction of activity-dependent Arc [2]. Arc mRNA is transported to the dendrites [3, 4, 6] perhaps via SUMOylation (reviewed in [7]), where it is intradendritically localized to activated synapses by phosphorylated ERK (extracellular signal-regulated kinase) signaling and actin polymerization [6, 8–11], translated into protein, and becomes a part of the postsynaptic junction [12]. The recruitment of Arc to the dendrites suggests its importance for synaptic plasticity that occurs after activation.

Arc expression has been strongly linked to long-term potentiation (LTP) and learning. High frequency stimulation (HFS) induces both LTP and Arc expression [3], which are dependent upon NMDA receptor activation [3, 4] but not upon the activation of AMPA receptors [12]. Additionally, intrahippocampal infusions of Arc antisense in vivo disrupt multiple aspects of LTP, indicating that Arc protein synthesis is required for the early expression, maintenance, and consolidation of enduring LTP ([13, 14], reviewed in [7]). In accordance with LTP as a molecular model for learning and memory, delivery of Arc antisense to the dorsal hippocampus produces long-term memory deficits in spatial water maze performance [13] and inhibitory avoidance in rats [15], indicating a necessary role for Arc protein in memory consolidation. Furthermore, Arc-knockout mice show impaired spatial learning in the Morris water maze task, disrupted fear memory to context and auditory stimuli, and deficits in conditioned taste aversion and object recognition [16]. Recent findings provide evidence for the role of Arc in the regulation of AMPA receptors through interactions with endocytic proteins in dendrites ([17, 18], reviewed in [19, 20]), as well as a function in the stabilization and the expansion of the F-actin cytoskeleton at activated synapses [14], strengthening the argument that Arc is involved in modifications that affect synaptic efficacy (reviewed in [7]).

 The protein product of the immediate early gene zif268 (also termed Egr1, or early growth response gene) is a transcription factor of the zinc finger family [21]. Expression of zif268 is regulated by synaptic activity and dependent upon NMDA receptor activation [22]. Induction of LTP produces increased expression of zif268 mRNA [21], and knockout of the zif268 gene in mice results in absent late LTP in the hippocampus and deficits in long-term memory for spatial water maze, conditioned taste aversion, socially transmitted food preference, and object recognition [23]. Additionally, infusions of zif268 antisense into the amygdala prior to contextual fear conditioning disrupt fear memory consolidation [24].

In the present set of experiments, we used Pavlovian fear conditioning to investigate the time-dependent expression of Arc and zif268. In Pavlovian fear conditioning, a neutral stimulus is paired with an aversive unconditional stimulus (UCS). Through this pairing, the once neutral stimulus becomes able to elicit a fear response (termed the conditional response, or CR). The animal also acquires fear for the context in which fear conditioning occurred. When the animal is presented with the shock-associated auditory stimulus or is placed back in the training context, it will exhibit fear behaviors indicating memory for the training experience.

The amygdala is crucial for the acquisition, consolidation, and expression of classically conditioned fear, as it receives information about both conditional and unconditional stimuli (CS and UCS, respectively) making it a site of associative convergence [25, 26]. Amygdala lesions and protein-synthesis inhibitors delivered to the amygdala disrupt fear conditioning [27–29]. The hippocampus is not necessary for conditional fear to an auditory CS in a delay paradigm, but it is essential for contextual fear. Post training hippocampal lesions abolish contextual fear in rodents, but they do not affect conditioning to an auditory stimulus [30] and protein-synthesis inhibitors given into the hippocampus block the acquisition of contextual fear memory [26, 31]. 

The present study examined the time course of Arc protein expression in the hippocampus following Pavlovian fear conditioning. In addition, the temporal profile of zif268, another plasticity-related gene product, was measured and compared to the pattern of expression for Arc protein. Immunohistochemistry followed western blot studies to show the localization of Arc and zif268 in hippocampal regions with elevated protein expression post training. Additional control groups for shock stimulation and simple exposure to auditory and contextual stimuli were analyzed with western blots to better determine the specific contribution of Arc and zif268 protein in the hippocampus.

## 2. Materials and Methods

### 2.1. Subjects

In all experiments, male Long Evans rats (*N* = 148; Harlan; Madison, WI) weighing approximately 350 g were used. The animals were housed individually with food and water *ad libitum*. The animal colony was climate-controlled and maintained on a 14 hr  :  10 hr light:dark cycle with lights on at 7:00 a.m. All experimental procedures were performed during the light cycle. All procedures were approved by the Institutional Animal Care and Use Committee at the University of Wisconsin-Milwaukee.

### 2.2. Apparatus

Four identical conditioning chambers were used for training. Each chamber was constructed from clear Plexiglas (front and back walls, ceiling) and stainless steel (side walls) and measured 28 × 20.5 × 21 cm (length × height × depth). Stainless steel rods spaced 12 mm apart served as the floor of the chamber and were used to deliver a mild footshock from a scrambled shock generator. Conditioning chambers were housed in sound-attenuating boxes that were illuminated by a 7.5 W white light bulb. There was a constant background noise of 56–60 dB produced by ventilation fans inside the boxes. The chambers were cleaned with 5% ammonium hydroxide solution between each rat. In all behavioral testing, the dependent measure was freezing behavior, which is operationally defined as the lack of all movement, except movement necessary for respiration [32]. The training procedure was recorded by video cameras installed inside the sound-attenuating chambers, and freezing behavior was scored by computer software (FreezeScan 1.0; Clever Sys. Inc; Reston, VA).

### 2.3. Behavioral Procedure

**Figure 1:**
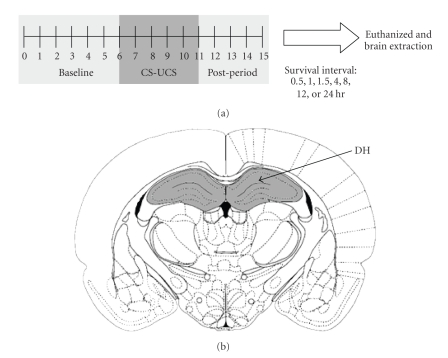
(a) Schematic depicting the fear conditioning procedure used. Rats were killed at varying time points following training and brain tissue was dissected for western blot analysis. (b) Frozen brain tissue was microdissected from the dorsal hippocampus. Shaded regions are representative of the size and location of tissue collected for western blots. CS: conditional stimulus, UCS: unconditional stimulus, DH: dorsal hippocampus.

Before any experimentation, rats were adapted to handling and transportation procedures for 3 min each on 6 consecutive days. Rats were trained in a single 15-min session of auditory-cued fear conditioning (Figure 1(a)). After an initial 6-min baseline period, the rats received four presentations of white noise (72 dB, 10 s) that coterminated with a footshock (1.3 mA, 1 s). These four presentations were separated by a 90-second intertrial interval. The rats remained in the chamber for an additional 4 min following the last footshock before being returned to their home cages. This training protocol has previously been shown to produce both contextual and auditory-cued fear memories [28, 29, 33]. Additional groups of animals were created to control separately for auditory and contextual experience and shock stimulation. One group experienced the same training protocol but with no shock stimuli delivered (WN-CXT), and another group received footshock immediately upon placement in the chamber and removed shortly afterward (SHK-only).

### 2.4. Western Blot Procedure

After training, animals were returned to their home cages and later euthanized with an overdose intraperitoneal injection of a sodium pentobarbital solution at varying time points post training. Animals were killed at 30 min, 60 min, 90 min, 4 hr, 8 hr, 12 hr, or 24 hr after training, and naïve home cage (HC) rats served as a control group. In a separate experiment, WN-CXT and SHK-only groups were euthanized 60 min after stimulus exposure and were compared to trained rats also killed 60-min post training as well as additional HC controls. It was not possible to control the animals' behavior (e.g., sleeping) during the survival interval, however, the occurrence of any such behaviors prior to euthanasia should be controlled for by the HC group. HC animals were killed at varying times during the day across the experiment to account for circadian patterns, unsystematic animal behavior, and any variation in the animal colony. Thus, the protein expression measured in the trained groups—beyond that observed in the HC animals—should be specific to the learning experience and not the result of unsystematic variability. 

Euthanized rats were decapitated and the brains were quickly removed, frozen on dry ice, and stored at −80°C. Tissue samples were microdissected from the dorsal hippocampus (Figure 1(b)). In all dissections, a rat brain atlas [34] and a rat brain matrix (Harvard Apparatus, Holliston, MA) were used to maintain consistency in tissue collection.

Hippocampal samples were homogenized manually with a pestle and glass tissue grinder in a buffer solution (all in 100 mL DDH_2_O: 0.605 g Tris-HCl, 0.25 g sodium deoxycholate, 0.876 g NaCl, 0.038 g EDTA, 0.0042 g NaF, 1 *μ*g/mL PMSF, 1 *μ*g/mL leupeptin, 1 *μ*g/mL aprotinin, 10 mL 10% SDS, 1 mM sodium orthovanadate) until there were no visible traces of solid matter. Homogenates were stored in centrifuge tubes and kept frozen at −80°C until time for centrifugation (4000 rpm for 20 min). The supernatant was removed, placed into small centrifuge tubes, and stored at −80°C. A Bradford protein assay was performed to determine the total amount of protein in the samples (Bio-Rad DC protein assay kit, Hercules, CA). Sample dilutions were pipetted into 96-well plates and compared to serial dilutions of the protein standard (Bio-Rad BSA 1.35 mg/mL) using a VersaMax plate reader and SoftMax Pro 4.3 LS software. Samples were discarded from further analysis if they did not meet a set criterion for variance, and this standard was applied to all groups equivalently.

Normalized protein samples were loaded onto 7.5% SDS gels for Arc blots or 9.0% SDS gels for zif268 blots using a Mini Protean holder filled with electrophoresis running buffer and a Bio-Rad PowerPac (200 V, 0.04 A constant, 90 min). Each experimental condition was represented on each gel to counterbalance any slight variation in blot development. Gels were washed in transfer buffer (3.03 g Tris, 14.4 g Glycine, 200 mL methanol, 5 mL 10% SDS, DDH_2_O up to 1 L) before the protein was transferred to PVDF membranes using a semidry transfer cell (Bio-Rad; PowerPac settings: 15 V constant, 2.00 A, 75 min). After protein transfer, membranes were incubated for 2 hr in blocking buffer (500 mL TBS, 15 g nonfat dry milk) and then exposed to primary antibody for 90–120 min. A monoclonal antibody for Arc protein (dilution 1  :  100 in antibody buffer, Santa Cruz), a polyclonal antibody for zif268 protein (dilution 1  :  1000, Cell Signaling), and a polyclonal antibody for *β*-actin (dilution 1  :  1000, Cell Signaling) were used in these experiments. After exposure to the primary antibody, membranes were washed twice for 15 min in antibody buffer (100 mL blocking buffer, 50 *μ*L Tween-20) before being incubated for 90–120 min in secondary antibody (goat anti-mouse for Arc blots—1  :  5000 dilution, Santa Cruz; goat anti-rabbit for zif268 and *β*-actin blots—1  :  2000 dilution, Upstate Biotechnology). Membranes were washed twice for 15 min in wash buffer (100 mL TBS, 50 *μ*L Tween-20) before exposure to chemiluminescence solution (Santa Cruz) for 3 min. Washes and incubations were generally done at room temperature, however, primary incubation was sometimes performed overnight at 4°C and then for 1 hr at room temperature. Developments were conducted in a dark room, where membranes with chemiluminescence were exposed to autoradiographic film in a cassette. Any disruptions in the signal during development of the films caused the sample to be excluded from further analysis.

The bands representing Arc (molecular weight (MW): 55 kDa), zif268 (MW: 75 kDa), and *β*-actin (MW: 45 kDa) were measured using densitometry software (NIH ImageJ). Optical density measures were computed for each hippocampal sample as a percentage of the home-cage animals' (control group) protein expression, and these results were statistically analyzed using one-way ANOVAs and Fisher's least significant difference (LSD) post-hoc comparisons when appropriate.

### 2.5. Immunofluorescence Procedure

Rats (*n* = 6) were euthanized with an overdose of isofluorane either 30 or 90 min after a single session of fear conditioning (15-min session with signaled shocks, see Figure 1(a)). The 30-min and 90-min time points were selected based on the time course for zif268 and Arc expression established by the western blot analysis. Two untrained HC animals were killed for a control group comparison. The rats were perfused transcardially with 0.1 M PBS followed by 10% buffered formaldehyde prior to decapitation. Brains were removed and placed in 10% buffered formaldehyde overnight, and then transferred to 30% sucrose formalin for cryoprotection for another 24 hr. Prior to slice collection, brains were rinsed 3 times in 0.1 M PBS for 10 min each. Using a freezing microtome, coronal slices (50-micron thick) were collected throughout the rostral-caudal extent of the dorsal hippocampus and placed into 24-well plates with 500 *μ*l of 0.1 M PBS. The slices were incubated on a titer plate in 1% sodium borohydride for 15 min, 0.1 M PBS twice for 10 min each, 10% normal goat serum for 30 min, and primary antibody for 30 min. Arc (1  :  100 dilution), zif268 (1  :  500 dilution), and NeuN (1  :  200 dilution) primary antibodies were used to determine the regional localization and neuronal colocalization of Arc and zif268 proteins. NeuN antibody (Chemicon-Millipore) binds to neuron-specific nuclear protein and is commonly used to identify neurons. Slices from each treatment condition were dual-labeled for Arc and zif268 protein to determine colocalization within individual neurons. The slices remained in primary antibody overnight at room temperature.

In the following day, all slices were incubated with two washes of 0.1 M PBS for 10 min each before incubation in antibody solution containing anti-mouse Alexa 488 and anti-rabbit Alexa 594 antibodies (1  :  500 dilution each, Invitrogen) for 2 hr in the dark. Slices were rinsed in two washes of 0.1M PBS for 5 min each. After incubation the slices were mounted onto unsubbed slides using Ultra Cruz mounting medium (Santa Cruz). Finally, the slides were coverslipped and sealed with a thin coat of nail polish around the edges. Slides were stored in the dark at −20°C until they were viewed. Photomicrographs were taken using a fluorescence microscope (Olympus). To determine coexpression of Arc and zif268 in the same neurons, separate images were taken of the same field for each protein and then merged using the DP Manager (Olympus). Arc-expressing neurons appear green in color (Alexa 488) and zif268-expressing neurons fluoresce red (Alexa 594), thus overlaying the images resulted in coexpressing neurons to appear yellow in color. The exposure time was the same for all the images collected, and any adjustments to the contrast or the brightness of the images were conducted exactly the same for relevant images.

### 2.6. Data Analysis

Protein expression values obtained from western blots were found to be normally distributed, thus the results were analyzed using parametric tests: one-way analysis of variance (ANOVA) followed by Fisher's LSD post-hoc comparisons when appropriate. In some cases, Pearson correlations were conducted on the normalized optical density values from western blots to investigate the relationship of protein expression in the dorsal hippocampus. SPSS statistical package was used in carrying out these analyses. Significance levels were set at *α* = 0.05, and data are presented as mean ± SEM.

## 3. Results

### 3.1. Acquisition of Conditional Fear

Seventy-six male Long Evans rats were trained in a single 15-min session of Pavlovian fear conditioning and later killed at varying time points post training (30 min, 60 min, 90 min, 4 hr, 8 hr, 12 hr, and 24 hr). All trained groups exhibited equivalent levels of freezing averaged across the 5-min period of CS-UCS presentations (*F*_(6,69)_ = 1.989, *P* > .05, data not shown).

### 3.2. Expression Profiles of Arc and Zif268 Proteins Differ in the Dorsal Hippocampus

Protein levels for each experimental condition were expressed as a percentage of the untrained HC animals' protein expression [% optical density (OD) of HC control]. Any animal with a %OD score more than 4 standard deviations from the mean was removed from further data analysis (Arc: no outliers; zif268: *n* = 1 from 24 hr group).

**Figure 2:**
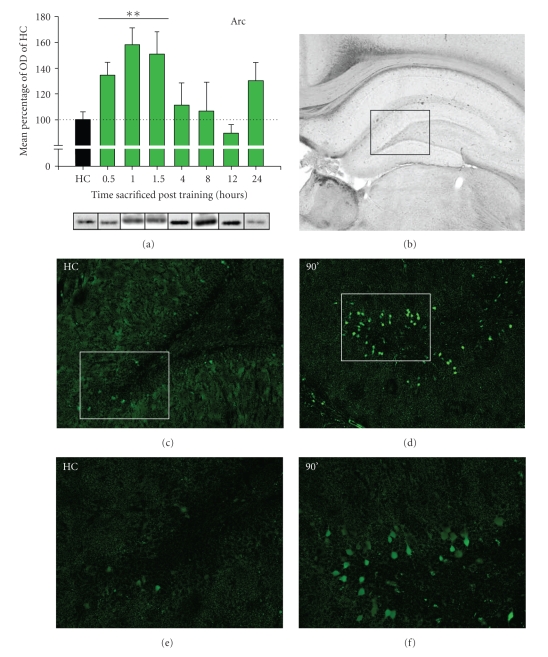
Expression of Arc protein in the dorsal hippocampus following fear conditioning. (a) The temporal expression profile for Arc protein shows a noticeable increase in expression at 30 min, continuing through 90 min post training. The rise in Arc protein diminishes over time, reaching basal levels by 4 hr post training. Bar graph represents the group means ± SEM. Representative western blot images are presented for each group directly below the corresponding graph plot. Significant differences from home cage (HC) controls are denoted with asterisks: *P < .05, **P < .01. (b) Brightfield photomicrograph (2× magnification) of the dorsal hippocampus. The boxed area indicates the region depicted in the immunofluorescence images of C and D (10× magnification). (c) Basal expression of Arc protein in the dentate gyrus is low in the home cage control animal. (d) Arc protein expression is significantly increased in granule cells of the dentate gyrus 90 min after fear conditioning. (e, f) Higher magnification photomicrographs (20× magnification) of the boxed regions from (c) and (d), respectively.

We found that induction of Arc protein expression for trained rats was monophasic in the dorsal hippocampus, with a significant increase detected between 30 min and 90 min post training before returning to near basal levels at 4 hr (Figure 2(a)). A one-way ANOVA revealed that the level of Arc protein expression was time-dependent following fear conditioning (*F*_(7,79)_ = 4.265, *P* < .001). LSD post hoc comparisons showed that when compared to untrained HC animals, Arc protein was significantly increased in trained rats at 30 min (*MD* = 34.4587, *P* < .01), 60 min (*MD* = 58.0035, *P* < .001), and 90 min (*MD* = 50.8044, *P* < .01). 

In order to determine the localization of Arc protein in the dorsal hippocampus, coronal brain slices were collected from rats killed at 90 min after training and prepared for immunohistochemistry. Increased Arc protein expression was observed in the granule cell layer of the dentate gyrus in the trained animals, whereas only sparse expression of Arc protein was detected in this same region in the unstimulated HC control rats (Figures 2(b)–2(f)). Arc-positive neurons were not evident in other regions of the dorsal hippocampus, such as the CA1. Although we did not quantify the Arc-positive neurons in the photomicrographs, the images suggest that the upregulation in Arc protein expression is primarily localized in the dentate gyrus.

**Figure 3:**
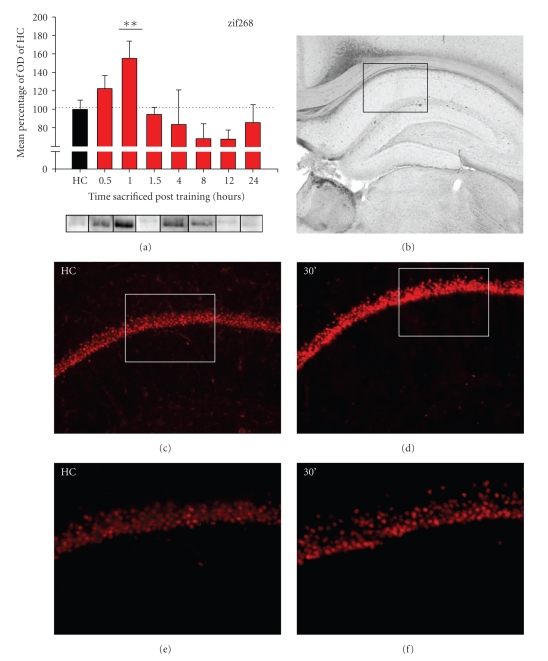
Expression of zif268 protein in the dorsal hippocampus following fear conditioning. (a) zif268 protein expression exhibits a noticeable increase at 30–60 min post-training. Statistical analysis revealed that protein levels are not different from HC after 60 min. Bar graph represents the group means + SEM. Representative western blot images are presented for each group directly below the corresponding graph. Significant differences from HC controls are denoted with asterisks: *P < .05, **P < .01. (b) Brightfield photomicrograph (2x magnification) of the dorsal hippocampus. The boxed area indicates the region of CA1 depicted in the immunofluorescence images of (c) and (d) (10x magnification). (c) Basal expression of zif268 protein in the CA1 of home cage animal. (d) zif268 protein expression is increased in CA1 neurons 30 min after fear conditioning. (e), (f) Higher magnification photomicrographs (20x magnification) of the boxed regions from (c) and (d), respectively.

A different temporal pattern was seen in the protein expression profile for zif268, with a single peak evident at 60 min post training (*F*_(7,72)_ = 3.228, *P* < .01, see Figure 3(a)). LSD post-hoc comparisons revealed that zif268 protein is significantly increased at 60 min when compared to HC animals (*MD* = 55.2942, *P* < .01). In fact, the protein level for zif268 measured at 60 min is significantly higher than all other time points (90 min: *MD* = 60.6474, *P* < .01; 4 hr: *MD* = 71.1209, *P* < .01; 8 hr: *MD* = 86.4159, *P* < .01; 12 hr: *MD* = 86.9798, *P* < .01; 24 hr: *MD* = 69.0759, *P* < .01) with the exception of 30 min (*MD* = 32.7638, *P* > .05). 

**Figure 4:**
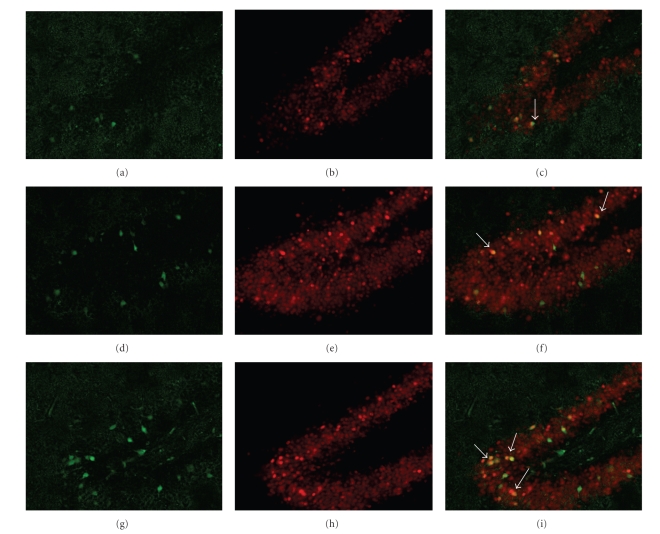
Photomicrographs (20x magnification) of neurons in the granule cell layer of the dentate gyrus expressing Arc and zif268 protein. Arc (green), zif268 (red), and merged images are shown for unstimulated home cage animals (a)–(c) and for animals killed 30 min (d)–(f) and 90 min (g)–(i) following fear conditioning. The number of Arc-positive neurons increased following fear conditioning (a), (d), (g), and the degree of zif268 immunoreactivity is greatest in the 30 min condition (e). Arc and zif268 are co-localized in dentate gyrus granule cells, which appear yellow when Arc and zif268 images are merged (c), (f), and (i). Example neurons co-expressing Arc and zif268 protein are indicated with arrows in the merged images.

To establish the localization of zif268 protein in the dorsal hippocampus, coronal brain slices from additional rats killed 30 min post training were collected and incubated with zif268 antibody. In agreement with findings from western blot analysis, conditioned rats showed qualitatively more expression of zif268 protein in the CA1 region of the dorsal hippocampus at 30 min after training, compared to HC controls (Figures 3(b)–3(f)). Similar increases were evident in the dentate gyrus but not in the CA3 region of the hippocampus (data not shown). Some transcription factors (such as zif268) have relatively high basal levels of expression [22], and this was observed in the unstimulated HC controls (Figures 3(c) and 3(e)). However, this basal level of zif268 expression was less than the level of zif268 protein induced behaviorally, as measured by western blots (Figure 3(a)) and captured visually in photomicrographs (Figures 3(d) and 3(f)). These images suggest that the increase in zif268 protein measured in western blot analysis is the result of upregulation of zif268 primarily in CA1 and the dentate gyrus (see Figure 4).

To validate the temporal changes observed for Arc and zif268 protein expression, the same hippocampal samples were assayed for *β*-actin, a constitutively expressed protein, using western blot analysis. The levels of *β*-actin protein in the trained groups did not show significant changes in expression compared to HC controls (*F*_(7,81)_ = 1.328, *P* > .05; data not shown), indicating that the upregulation in Arc and zif268 protein is unique and specific to the behavioral training experience.

In summary, the expression profile for these proteins demonstrates an early increase in zif268 expression at 30–60 min post training, followed by a gradual monophasic wave in Arc induction lasting through 90 min (Figures 2 and 3). The temporal dynamics of these proteins are distinctive and reflect the difference in the functions for Arc and zif268 (i.e., synapse-specific changes and transcriptional regulation, respectively). The delay between the peaks for zif268 and Arc proteins corroborates previous research showing that Arc is a transcriptional target of zif268 [35] and that multiple genomic responses are activated as a consequence of fear acquisition.

### 3.3. Correlations between Arc and Zif268 Protein Expression

Using Pearson correlation on the western blot optical density values, we investigated the relationship between expression of Arc and zif268 proteins in the dorsal hippocampus. Correlational analysis indicated a direct moderate relationship between the levels of these proteins in the dorsal hippocampus after fear conditioning (*r*_(79)_ = 0.244, *P* < .05). Upon closer inspection, we discovered that this relationship was driven by the positive correlation between Arc and zif268 proteins in the animals killed 30 min after training (*r*_(15)_ = 0.510, *P* < .05), as significant correlations between these proteins were not found for the other experimental conditions. Other researchers have found Arc and zif268 mRNA expression in the hippocampus to be correlated following training in hippocampus-dependent tasks, such as the spatial water maze [36, 37]. 

To investigate the colocalization between Arc and zif268 proteins in the hippocampus, brain slices collected from trained rats (sampled 30 and 90 min post training) and from naïve HC rats were dual labeled for these proteins and viewed using epifluorescence microscopy. In the unstimulated HC animals, only a few neurons in the dentate gyrus expressed Arc protein (Figure 4(a)); however, many of these neurons were zif268-positive (Figure 4(b)). The merged image revealed that the Arc-positive neurons in the dentate gyrus also expressed zif268 (Figure 4(c)). In the trained rats, more neurons expressed Arc protein, with maximal number of Arc-positive neurons reached at 90 min (Figures 4(d) and 4(g)). Further, many more neurons in the dentate gyrus expressed zif268, with qualitatively more zif268-positive neurons shown at 30 min (Figure 4(e)) compared to slices collected from naïve and 90-min animals (Figures 4(b) and 4(h)). As was seen in the naïve HC animals, Arc and zif268 proteins expressed in trained animals are co-localized in the same neurons as depicted in the merged images (Figures 4(f) and 4(i)). These findings qualitatively replicate the western blot analysis results showing increased Arc at 90 min and zif268 around 30 min post training in the dorsal hippocampus. They also extend those findings to suggest that after fear conditioning co-expression of zif268 and Arc protein increases.

### 3.4. Arc and Zif268 Protein Expression in the Hippocampus Is Driven by Behavioral Experience and Associative Learning

To determine if the time-dependent changes in hippocampal Arc and zif268 protein expression were specific to associative learning, rats were assigned to one of four conditions: trained (T; *n* = 14), white noise and context exposure (WN-CXT; *n* = 9), immediate shock (SHK-only; *n* = 8), or unstimulated home cage control (HC; *n* = 12). Trained animals received the same fear conditioning protocol used previously (Figure 1(a)). WN-CXT animals experienced the same training paradigm but with the shock generator turned off, and SHK-only rats received shock upon placement in the chamber and then were immediately removed. All rats were killed 60 min post-experience and dorsal hippocampal tissue was processed for western blots. Protein levels for each experimental condition were expressed as a percentage of the untrained HC animals' protein expression [% optical density (OD) of HC control]. 

**Figure 5:**
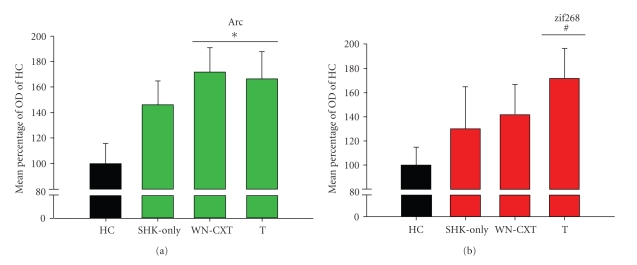
Expression of Arc and zif268 protein in the dorsal hippocampus is induced by context exposure and associative learning, respectively. (a) Rats exposed to auditory fear conditioning (T) or contextual and auditory stimuli without shock (WN-CXT) show similar increases in Arc protein relative to unstimulated home cage (HC) controls. (b) Auditory fear conditioning produced increased zif268 protein expression in the dorsal hippocampus in comparison to HC animals. WN-CXT and SHK-only groups did not show significant changes from basal levels. Bar graphs represent the group means ± SEM. *P < .05 for ANOVA. ^#^ *P* < .05 for planned comparisons.

Arc protein levels did change significantly due to behavioral experience in the training context (Figure 5(a); (*F*_(3,33)_ = 3.007, *P* < .05)). Specifically, both the trained and WN-CXT groups had increased levels of Arc protein compared to unstimulated HC controls (Trained: *MD* = 66.3183, *P* < .05; WN-CXT: *MD* = 71.6449, *P* < .05), which was not observed in the SHK-only condition (*MD* = 46.0808, *P* > .05). The expression of Arc protein in the trained group was similar to Arc protein levels observed in the 1-hr group measured in the original time course (~60% increase; Figures 2(a) and 5(a)).

A different pattern of protein expression was measured for zif268, where marked increases were seen only in the trained condition (Figure 5(b)). The overall ANOVA between HC, SHK-only, WN-CXT, and trained groups did not meet our statistical criterion (*F*_(3,33)_ = 1.593, *P* > .05). However, the level of zif268 in the trained group was similar to that observed in the 1-hr time group of the original time course (~65%; Figures 3(a) and 5(b)). Therefore, we conducted a linear planned comparison between the trained and HC groups which did indicate a significant upregulation of zif268 protein in the trained group (*F*_(1,33)_ = 4.693, *P* < .05). 

In contrast to both Arc and zif268 protein expression, there were no significant changes in protein levels in the loading control *β*-actin across the conditions (*F*_(3,37)_ = 0.112, *P* > .05; data not shown).

## 4. Discussion

The effector IEG Arc has been implicated in synaptic plasticity underlying learning and memory. Our aim was to extend these earlier findings by investigating the time-dependent expression of Arc protein induced by Pavlovian fear conditioning and compare it to the expression profile of another IEG, zif268. We found Arc protein to be expressed soon after fear conditioning in the dorsal hippocampus. Gradual increases in Arc protein were detected by 30 min, and the single peak in the expression profile emerged at 1-2 hr post training before returning to baseline levels at 4 hr. Arc protein was primarily localized in the granule cell layer of the dentate gyrus. These data indicate that Arc protein expression induced in the dorsal hippocampus by fear conditioning is time-dependent and monophasic. Arc protein is likely involved in the consolidation of contextual fear memories supported by the hippocampus, since auditory delay fear conditioning is not reliant on the hippocampus [26, 30].

The levels of Arc protein expression in the dorsal hippocampus were positively correlated with the regulatory transcription factor IEG zif268. The expression profile for zif268 in the dorsal hippocampus was also monophasic; however, maximal protein levels were measured at 60 min after fear conditioning, with increased expression seemingly localized to the dentate gyrus and CA1.

In a separate experiment, we investigated if the induction of these proteins was specific to associative learning or was the result of behavioral experience more generally. Arc protein expression increased in rats that were trained as well as in those animals exposed to the context and auditory stimuli. Delivery of immediate shock did not produce a significant increase in Arc, so Arc protein expression is not linked to UCS exposure per se. Further, we would not predict a significant learning-related increase for the immediate shock condition as this procedure does not result in normal learning. Since both training and exposure to the training chamber induced similar levels of Arc expression, this effect likely relates to contextual processing. Similar alterations in Arc mRNA in the hippocampus have been observed using catFISH analysis for contextual fear conditioned and context-exposed animals [38]. In contrast, zif268 appeared to be significantly upregulated only in trained rats. This selective increase in hippocampal zif268 protein only in the trained group and not in SHK-only and WN-CXT conditions is similar to the upregulation of zif268 observed during retrieval of a context fear memory relative to cued-fear retrieval or reexposure to a context not paired with shock [39]. The significance of this difference from the pattern seen with Arc protein is not yet clear, but perhaps zif268 expression in the hippocampus is more specifically related to the formation of aversive memories.

The single phase of Arc protein upregulation we observed is similar to forskolin- and ECS- (electroconvulsive shock) induced expression of Arc mRNA [1, 3, 4, 40] and protein [1, 41], with increased expression measured 30 min to 4 hr post activation and a return to basal levels by 8 or 24 hr. Our data are also in accordance with recent LTP in vivo investigations, in which Arc antisense oligodeoxynucleotide applied 2 hr, but not 4 hr, post-LTP induction resulted in the reversal of LTP, suggesting that the role of Arc protein is time-limited [14]. Furthermore, our hippocampal data complement findings from other behavioral paradigms. For example, Arc mRNA expression in the hippocampus following spatial water maze training peaks at 30 min post training and returns to baseline at 6 hr [37]. We do detect elevated levels of Arc protein at 30 min, which may be the product of existing or newly transcribed mRNA, a possibility we have not yet investigated or found answered in the literature. However, Arc protein at 1-2 hr is likely translated from new transcripts synthesized as a consequence of the training experience. This hypothesis is congruent with data suggesting a very rapid turnover of Arc, on the scale of minutes to a few hours [42, 43]. Although spatial water maze and contextual fear conditioning are two different hippocampus-dependent tasks, the mRNA and protein time courses for these two studies produce a logical sequence when combined, such that a peak increase for Arc mRNA at 30 min is followed by maximal Arc protein levels at 90 min.

Multiple waves of increased protein levels may follow a training experience, and the number of phases depends upon the training parameters used [44, 45]. For instance, biphasic expression has been measured for other proteins in response to fear conditioning [45]; however, our data suggest that the expression of Arc and zif268 protein in the hippocampus is monophasic following acquisition of conditioned fear. Monophasic expression of Arc is not necessarily true for all behavioral paradigms, as Ramirez-Amaya et al. found Arc protein to be expressed in two phases in CA1 and CA3 of the hippocampus—first phase at 30 min to 2 hr and the second phase at 8 and 24 hr—following a single exploration session [46]. In the same study, they found a single wave of Arc protein in the dentate gyrus that lasted up to 8 hr post exploration, which is similar to the primarily monophasic expression of Arc protein in the dentate gyrus reported here [46].

Although the increase was not significant, Arc protein did seem to show a moderate increase 24 hr after training. Since these animals were killed at varying times during the day, we can conclude that this increase is not due to set circadian patterns in IEG expression. However, memory does seem to have a time-of-day dependence, such that testing at a similar time of day as when training occurred produces better recall. Recently, some attention has been given to exploring clock-genes in structures such as the hippocampus that may influence the expression of other proteins and may in effect create time-of-day dependence in memory [47]. Circadian fluctuation in protein phosphorylation has been previously observed in the hippocampus for MAPK [48], and this signaling pathway has been implicated in Arc translation [49]. Whether Arc or any IEG is regulated in such a manner is purely speculation at this point, but certainly it is an important consideration for understanding time-dependent changes in protein translation. 

The post training expression of Arc and zif268 proteins in the dorsal hippocampus corresponds to the time window for protein synthesis-dependent memory consolidation. The transient nature of Arc expression after training may be related to evidence indicating that Arc mRNA is targeted for nonsense-mediated mRNA decay (NMD) as this gene contains two conserved 3′-UTR introns ([50], reviewed in [7, 19]). NMD is a translation-dependent decay mechanism that likely halts protein expression to produce finite protein levels that are temporally specific to the learning event. Along with other degradation pathways, such as ubiquitin-dependent degradation by proteasomes, NMD probably restricts the protein composition to local activated synapses. 

The notion that the burst of Arc protein expression is temporally linked to the learning event is further supported by studies reporting that the level of training-induced Arc expression in the hippocampus is coupled with learning performance. For example, Guzowski and colleagues demonstrated that the amount of hippocampal Arc mRNA was positively correlated with an animal's performance in hippocampus-dependent water maze learning [37]. Furthermore, the same study revealed hippocampal Arc mRNA expression to be correlated with the spatial water maze task (hippocampus-dependent) but not with the nonspatial water maze task (hippocampus-independent), indicating that Arc expression is associated specifically with learning experiences. Presumably, the increased induction of Arc and zif268 proteins measured in the present study is the result of contextual processing and associative fear learning, respectively, as the dorsal hippocampus is important for the acquisition and initial consolidation of contextual fear memory.

Recent investigations on the molecular pathways leading to the induction of Arc have focused on brain-derived neurotrophic factor (BDNF), ERK, and cAMP-PKA activation. Converging evidence indicates that Arc is a downstream effector of BDNF activation [14, 43], and PKA-dependent Arc protein expression can be stimulated either through the activation of NMDA receptors or G_s_-coupled dopamine or *β*-adrenergic receptors [41, 51]. Recent work by Bramham and colleagues suggests that the initiation of Arc translation is the result of ERK-MNK (extracellular signal-regulated kinase-mitogen-activated protein kinase-interacting kinase) signaling in the dentate gyrus, a structure where we observed increases in Arc protein [49]. Pharmacological blockade of ERK with the MEK inhibitor U0126 abolishes LTP and Arc protein expression; however, similar results are not observed when mTORC1 (mammalian target of rapamycin complex 1) signaling is inhibited by the application of the protein-synthesis inhibitor rapamycin [49]. 

Comparatively, less is known about the interaction of zif268 and Arc following synaptic activation. Zif268 is a transcriptional regulator, and it is noteworthy that in our study the peak in zif268 expression at 60 min occurs just prior to the peak in Arc at 90 min. The time course for these proteins presented here corroborates earlier evidence that Arc is a transcriptional target of zif268, as the Arc promoter has a functional ERE (Egr response element) [35]. Additionally, we showed that the levels of hippocampal Arc and zif268 were correlated with one another following fear conditioning, which further supports the functional relationship between these two IEG products. Work by others similarly found Arc and zif268 mRNA to be upregulated following spatial exploration, and these IEGs are often detected in the same nucleus [36]. These mRNAs are correspondingly increased in the hippocampus following spatial water maze training, and their expression profiles are positively correlated within this brain structure [37]. However, the relationship between Arc and zif268 is not perfect, as Arc protein increases we measured at 30 min are likely not the result of zif268 regulation and are believed to be induced by one of the other pathways implicated in Arc expression. Further research is needed to determine what role zif268 has in the induction of Arc that contributes to synaptic modifications underlying long-term memory. Our data suggest that these two proteins interact soon after a learning experience, most likely orchestrating postsynaptic changes to increase synaptic efficacy and support memory formation. 

## 5. Conclusions

In summary, Arc and zif268 proteins are transiently increased in the dorsal hippocampus in a manner that suggests that these two proteins work together to support contextual learning in fear conditioning. Although we did not establish a causal relationship between associative learning and Arc and zif268 protein expression, we have shown that these IEGs are consistently upregulated in the hippocampus during the period when memory for context is being consolidated. The time frame of behaviorally-induced Arc and zif268 protein in the dorsal hippocampus corresponds to a critical time window in which protein synthesis is required for memory consolidation. Further, immunostaining revealed an increase in expression of Arc and zif268 protein in the same hippocampal neurons after fear conditioning, suggesting a relationship between Arc and zif268 colocalization in consolidation of a contextual fear memory.

## References

[1] Wallace CS, Lyford GL, Worley PF, Steward O (1998). Differential intracellular sorting of immediate early gene mRNAs depends on signals in the mRNA sequence. *Journal of Neuroscience*.

[2] Kawashima T, Okuno H, Nonaka M (2009). Synaptic activity-responsive element in the *Arc/Arg3.1* promoter essential for synapse-to-nucleus signaling in activated neurons. *Proceedings of the National Academy of Sciences of the United States of America*.

[3] Link W, Konietzko U, Kauselmann G (1995). Somatodendritic expression of an immediate early gene is regulated by synaptic activity. *Proceedings of the National Academy of Sciences of the United States of America*.

[4] Lyford GL, Yamagata K, Kaufmann WE (1995). Arc, a growth factor and activity-regulated gene, encodes a novel cytoskeleton-associated protein that is enriched in neuronal dendrites. *Neuron*.

[5] Vazdarjanova A, Ramirez-Amaya V, Insel N (2006). Spatial exploration induces ARC, a plasticity-related immediate-early gene, only in calcium/calmodulin-dependent protein kinase II-positive principal excitatory and inhibitory neurons of the rat forebrain. *Journal of Comparative Neurology*.

[6] Rodriguez JJ, Davies HA, Silva AT (2005). Long-term potentiation in the rat dentate gyrus is associated with enhanced Arc/Arg3.1 protein expression in spines, dendrites and glia. *European Journal of Neuroscience*.

[7] Bramham CR, Alme MN, Bittins M (2010). The Arc of synaptic memory. *Experimental Brain Research*.

[8] Huang F, Chotiner JK, Steward O (2007). Actin polymerization and ERK phosphorylation are required for Arc/Arg3.1 mRNA targeting to activated synaptic sites on dendrites. *Journal of Neuroscience*.

[9] Moga DE, Calhoun ME, Chowdhury A, Worley P, Morrison JH, Shapiro ML (2004). Activity-regulated cytoskeletal-associated protein is localized to recently activated excitatory synapses. *Neuroscience*.

[10] Steward O, Wallace CS, Lyford GL, Worley PF (1998). Synaptic activation causes the mRNA for the leg Arc to localize selectively near activated postsynaptic sites on dendrites. *Neuron*.

[11] Steward O, Worley PF (2001). A cellular mechanism for targeting newly synthesized mRNAs to synaptic sites on dendrites. *Proceedings of the National Academy of Sciences of the United States of America*.

[12] Steward O, Worley P (2002). Local synthesis of proteins at synaptic sites on dendrites: role in synaptic plasticity and memory consolidation?. *Neurobiology of Learning and Memory*.

[13] Guzowski JF, Lyford GL, Stevenson GD (2000). Inhibition of activity-dependent arc protein expression in the rat hippocampus impairs the maintenance of long-term potentiation and the consolidation of long-term memory. *Journal of Neuroscience*.

[14] Messaoudi E, Kanhema T, Soule J (2007). Sustained Arc/Arg3.1 synthesis controls long-term potentiation consolidation through regulation of local actin polymerization in the dentate gyrus in vivo. *Journal of Neuroscience*.

[15] McIntyre CK, Miyashita T, Setlow B (2005). Memory-influencing intra-basolateral amygdala drug infusions modulate expression of Arc protein in the hippocampus. *Proceedings of the National Academy of Sciences of the United States of America*.

[16] Plath N, Ohana O, Dammermann B (2006). Arc/Arg3.1 is essential for the consolidation of synaptic plasticity and memories. *Neuron*.

[17] Chowdhury S, Shepherd JD, Okuno H (2006). Arc/Arg3.1 interacts with the endocytic machinery to regulate AMPA receptor trafficking. *Neuron*.

[18] Shepherd JD, Rumbaugh G, Wu J (2006). Arc/Arg3.1 mediates homeostatic synaptic scaling of AMPA receptors. *Neuron*.

[19] Bramham CR, Worley PF, Moore MJ, Guzowski JF (2008). The immediate early gene Arc/Arg3.1: regulation, mechanisms, and function. *Journal of Neuroscience*.

[20] Tzingounis AV, Nicoll RA (2006). Arc/Arg3.1: linking gene expression to synaptic plasticity and memory. *Neuron*.

[21] Cole AJ, Saffen DW, Baraban JM, Worley PF (1989). Rapid increase of an immediate early gene messenger RNA in hippocampal neurons by synaptic NMDA receptor activation. *Nature*.

[22] Worley PF, Christy BA, Nakabeppu Y, Bhat RV, Cole AJ, Baraban JM (1991). Constitutive expression of zif268 in neocortex is regulated by synaptic activity. *Proceedings of the National Academy of Sciences of the United States of America*.

[23] Jones MW, Errington ML, French PJ (2001). A requirement for the immediate early gene Zif268 in the expression of late LTP and long-term memories. *Nature Neuroscience*.

[24] Malkani S, Wallace KJ, Donley MP, Rosen JB (2004). An egr-1 (zif268) antisense oligodeoxynucleotide infused into the amygdala disrupts fear conditioning. *Learning and Memory*.

[25] Fanselow MS, LeDoux JE (1999). Why we think plasticity underlying pavlovian fear conditioning occurs in the basolateral amygdala. *Neuron*.

[26] Helmstetter FJ, Parsons RG, Gafford GM (2008). Macromolecular synthesis, distributed synaptic plasticity, and fear conditioning. *Neurobiology of Learning and Memory*.

[27] Bailey DJ, Sun W, Thompson RF, Kim JJ, Helmstetter FJ (1999). Acquisition of fear conditioning in rats requires the synthesis of mRNA in the amygdala. *Behavioral Neuroscience*.

[28] Parsons RG, Gafford GM, Baruch DE, Riedner BA, Helmstetter FJ (2006). Long-term stability of fear memory depends on the synthesis of protein but not mRNA in the amygdala. *European Journal of Neuroscience*.

[29] Parsons RG, Gafford GM, Helmstetter FJ (2006). Translational control via the mammalian target of rapamycin pathway is critical for the formation and stability of long-term fear memory in amygdala neurons. *Journal of Neuroscience*.

[30] Kim JJ, Fanselow MS (1992). Modality-specific retrograde amnesia of fear. *Science*.

[31] Fischer A, Sananbenesi F, Schrick C, Spiess J, Radulovic J (2004). Distinct roles of hippocampal de novo protein synthesis and actin rearrangement in extinction of contextual fear. *Journal of Neuroscience*.

[32] Blanchard RJ, Blanchard DC (1969). Crouching as an index of fear. *Journal of Comparative and Physiological Psychology*.

[33] Gafford GM, Parsons RG, Helmstetter FJ (2005). Effects of post-training hippocampal injections of midazolam on fear conditioning. *Learning and Memory*.

[34] Paxinos G, Watson C (1998). *The Rat Brain in Stereotaxic Coordinates*.

[35] Li L, Carter J, Gao X, Whitehead J, Tourtellotte WG (2005). The neuroplasticity-associated Arc gene is a direct transcriptional target of early growth response (Egr) transcription factors. *Molecular and Cellular Biology*.

[36] Guzowski JF, McNaughton BL, Barnes CA, Worley PF (1999). Environment-specific expression of the immediate-early gene Arc in hippocampal neuronal ensembles. *Nature Neuroscience*.

[37] Guzowski JF, Setlow B, Wagner EK, McGaugh JL (2001). Experience-dependent gene expression in the rat hippocampus after spatial learning: a comparison of the immediate-early genes Arc, c-fos, and zif268. *Journal of Neuroscience*.

[38] Barot SK, Chung A, Kim JJ, Bernstein IL (2009). Functional imaging of stimulus convergence in amygdalar neurons during Pavlovian fear conditioning. *PLoS One*.

[39] Hall J, Thomas KL, Everitt BJ (2001). Cellular imaging of zif268 expression in the hippocampus and amygdala during contextual and cued fear memory retrieval: selective activation of hippocampal CA1 neurons during the recall of contextual memories. *Journal of Neuroscience*.

[40] Larsen MH, Olesen M, Woldbye DPD (2005). Regulation of activity-regulated cytoskeleton protein (Arc) mRNA after acute and chronic electroconvulsive stimulation in the rat. *Brain Research*.

[41] Bloomer WAC, VanDongen HMA, VanDongen AMJ (2008). Arc/Arg3.1 translation is controlled by convergent *N*-methyl-D-aspartate and G_s_-coupled receptor signaling pathways. *Journal of Biological Chemistry*.

[42] Rao VR, Pintchovski SA, Chin J, Peebles CL, Mitra S, Finkbeiner S (2006). AMPA receptors regulate transcription of the plasticity-related immediate-early gene Arc. *Nature Neuroscience*.

[43] Yin Y, Edelman GM, Vanderklish PW (2002). The brain-derived neurotrophic factor enhances synthesis of Arc in synaptoneurosomes. *Proceedings of the National Academy of Sciences of the United States of America*.

[44] Bourtchouladze R, Abel T, Berman N, Gordon R, Lapidus K, Kandel ER (1998). Different training procedures recruit either one or two critical periods for contextual memory consolidation, each of which requires protein synthesis and PKA. *Learning and Memory*.

[45] Trifilieff P, Herry C, Vanhoutte P (2006). Foreground contextual fear memory consolidation requires two independent phases of hippocampal ERK/CREB activation. *Learning and Memory*.

[46] Ramirez-Amaya V, Vazdarjanova A, Mikhael D, Rosi S, Worley PF, Barnes CA (2005). Spatial exploration-induced Arc mRNA and protein expression: evidence for selective, network-specific reactivation. *Journal of Neuroscience*.

[47] Jilg A, Lesny S, Peruzki N, Schwegler H, Selbach O, Dehghani F, Stehle JH (2010). Temporal dynamics of mouse hippocampal clock gene expression support memory processing. *Hippocampus*.

[48] Eckel-Mahan KL, Phan T, Han S (2008). Circadian oscillation of hippocampal MAPK activity and cAMP: implications for memory persistence. *Nature Neuroscience*.

[49] Panja D, Dagyte G, Bidinosti M (2009). Novel translational control in Arc-dependent long term potentiation consolidation in vivo. *Journal of Biological Chemistry*.

[50] Giorgi C, Yeo GW, Stone ME (2007). The EJC factor eIF4AIII modulates synaptic strength and neuronal protein expression. *Cell*.

[51] Waltereit R, Dammermann B, Wulff P (2001). Arg3.1/Arc mRNA induction by Ca^2+^ and cAMP requires protein kinase a and mitogen-activated protein kinase/extracellular regulated kinase activation. *Journal of Neuroscience*.

